# Health-economic evaluation of home telemonitoring for COPD in Germany: evidence from a large population-based cohort

**DOI:** 10.1007/s10198-016-0834-x

**Published:** 2016-10-03

**Authors:** Dmitrij Achelrod, Jonas Schreyögg, Tom Stargardt

**Affiliations:** Hamburg Center for Health Economics (HCHE), Universität Hamburg, Esplanade 36, 20354 Hamburg, Germany

**Keywords:** Telemonitoring, COPD, Cost-effectiveness, Administrative data, I18, H51

## Abstract

**Introduction:**

Telemonitoring for COPD has gained much attention thanks to its potential of reducing morbidity and mortality, healthcare utilisation and costs. However, its benefit with regard to clinical and economic outcomes remains to be clearly demonstrated.

**Objective:**

To analyse the effect of Europe’s largest COPD telemonitoring pilot project on direct medical costs, health resource utilisation and mortality at 12 months.

**Methods:**

We evaluated a population-based cohort using administrative data. Difference-in-difference estimators were calculated to account for time-invariant unobservable heterogeneity after removing dissimilarities in observable characteristics between the telemonitoring and control group with a reweighting algorithm.

**Results:**

The analysis comprised 651 telemonitoring participants and 7047 individuals in the standard care group. The mortality hazards ratio was lower in the intervention arm (HR 0.51, 95 % CI 0.30–0.86). Telemonitoring cut total costs by 895 € (*p* < 0.05) compared to COPD standard care, mainly driven by savings in COPD-related hospitalisations in (very) severe COPD patients (−1056 €, *p* < 0.0001). Telemonitoring enrolees used healthcare (all-cause and COPD-related) less intensely with shorter hospital stays, fewer inpatient stays and smaller proportions of people with emergency department visits and hospitalisations (all *p* < 0.0001). Reductions in mortality, costs and healthcare utilisation were greater for (very) severe COPD cases.

**Conclusion:**

This is the first German study to demonstrate that telemonitoring for COPD is a viable strategy to reduce mortality, healthcare costs and utilisation at 12 months. Contrary to widespread fear, reducing the intensity of care does not seem to impact unfavourably on health outcomes. The evidence offers strong support for introducing telemonitoring as a component of case management.

## Introduction

Chronic obstructive pulmonary disease (COPD) is an inflammatory disease of the respiratory system and is aggravated by acute respiratory exacerbations and systemic comorbidities. COPD causes elevated mortality and morbidity as well as soaring healthcare expenditure and utilisation [1, 2]. The number of individuals with COPD in Germany will grow from 5.9 [3] to 8.0 [4] million by 2050 while COPD is expected to become the world’s fourth most common cause of death within the next decade [5]. In search of cost-effective concepts of chronic care management, researchers and policy-makers have increasingly recognised the potential of telemedicine in reducing morbidity and mortality, as well as healthcare utilisation and its associated costs [6]. In particular, home telemonitoring (TM)—a technology measuring patients’ clinical parameters/symptoms [e.g. forced expiratory volume in one second (FEV_1_), oxygen saturation, sputum] at home and allowing communication between healthcare professionals and patients over distance—has gained much attention. Practitioners expect that telemonitoring can anticipate unscheduled, COPD-related physician/emergency department (ED) visits and hospitalisations by detecting anomalies in patients’ vital signs sufficiently early.

However, despite a growing body of evidence for TM in the management of COPD and other chronic diseases, such as congestive heart failure (CHF), the benefit of telemonitoring with regard to clinical and economic outcomes remains to be clearly demonstrated [6, 7]. Meta-analyses indicate that telemonitoring reduces the odds ratio of all-cause hospitalisation and ED visits by up to 54 % [8–10] and 73 % [8, 10], respectively, but has no impact on hospital length of stay, disease-specific quality of life (QoL) or mortality [8–11]. Most studies did not differentiate between COPD-related and all-cause healthcare use, leaving space for speculation about the effect on respiratory-related resource utilisation. Similarly, the evidence on cost-effectiveness is very meagre and inconclusive [9]. Recent cost-utility analyses from the UK found that telemonitoring was very unlikely to be cost-effective, with an incremental cost-effectiveness ratio (ICER) ranging between ~120,000 € [12] and ~178,000 € per quality-adjusted life year (QALY) gained [13]. In contrast, a modelling study in the German context found telemonitoring to be cost-effective (ICER 15,400 €) [14].

These findings need to be interpreted with caution though, and their applicability to the German context cannot be warranted because of the complete absence of German studies. The few telemonitoring interventions evaluated were highly heterogeneous, employing manifold technologies that ranged between simplistic telephone calls, patient education, virtual video-consultations, semi-automated transmission of vital parameters or a combination thereof [8]. The breadth and frequency of parameter measurements as well as availability and qualification of support staff diverged across studies. Short follow-up periods (range 2–12 months, mode 6 months) precluded statements about long-term effectiveness [9] and studies were typically under-powered [7] due to small sample sizes (range 18 [10] to 256 [11], median 70 [11]). Moreover, most studies were controlled trials and thus conducted in a well-ordered clinical environment that might lack comparability to routine care settings.

Given the dearth of much-needed evidence, the aim of this study is to analyse the effect of Germany’s largest COPD telemonitoring pilot project on direct medical costs, health resource utilisation and mortality. The intervention consisted of a telemonitoring set for transmitting vital parameters, clinical support and patient education. We estimate incremental costs and effectiveness by comparing a COPD telemonitoring and a COPD standard care cohort over a period of 1 year. In doing so, we address the limitations of existing studies in numerous ways. First, to the best knowledge of the authors, this is so far the largest evaluation of COPD telemonitoring in Europe. A follow-up period of 1 year in conjunction with a sample size that exceeds the mean sample size of conducted RCTs by a factor of ten enables measuring mid-term outcomes reliably. Second, we investigate the incremental causal effect of telemonitoring in pragmatic, routine clinical settings by using a combination of entropy balancing and difference-in-difference estimators. By isolating COPD-related from all-cause outcomes, we can make precise judgements about the effectiveness of telemonitoring on respiratory-related outcomes. Finally, we consider incremental costs in addition to effectiveness of the intervention, and we are the first to conduct an evaluation of telemonitoring for COPD in Germany.

## Methods

### Study design and study sample

Costs from the sickness fund perspective and effectiveness of telemonitoring were evaluated in an observational, retrospective, population-based cohort study design. We compared outcomes of patients receiving telemonitoring in addition to standard care with those of a cohort only receiving standard care over a period of 12 months. The analysis was based on administrative data from AOK Bayern (4.4 million insurances in 2014) which is Germany’s fourth largest sickness fund. The dataset contained longitudinal patient-level information on socio-demographic status, medical diagnoses, direct medical costs, as well as on healthcare utilisation between 2009 and 2014.

Patients (>18 years of age) with COPD were required (a) to have had an in- or outpatient ICD-GM-10 (J44) diagnosis in the dataset of the sickness fund and (b) to having been hospitalised with a COPD or COPD-related diagnosis (ICD J41–J44) within 24 months before the index date (variable date for telemonitoring group; 1 January 2013 for control group). The patient cohort was subsequently divided into an intervention group, i.e. patients that voluntarily enrolled in the telemonitoring programme for the first time between November 2012 and December 2013, and a control group, i.e. patients that had never been members of the telemonitoring programme at any point in time between 2009 and 2014. For telemonitoring enrolees, outcomes were measured for 12 months starting from their individual telemonitoring enrolment (index date between November 2012 and December 2013), while for the control group, outcomes were assessed in the 12-month period starting from their common index date (1 January 2013).

In order to allow for risk adjustment, we stipulated a period of 2 years prior to the index date (variable date for telemonitoring group; 1 January 2013 for control group) as the basis for determining patient-level risk profiles. Applying equally to the telemonitoring and control group, individuals were excluded from this study if they (1) switched between the telemonitoring and control group, (2) had not been constantly enrolled at the sickness fund during the 2-year risk adjustment, or (3) the 1-year observation period. Patients who died during the observation period were not excluded. Individuals were excluded if they were suffering from predefined diseases [malignant neoplasms (ICD C00–C97), moderate/severe intellectual disabilities (ICD F71–F74, F78), Parkinson’s (ICD G20–G23) and Alzheimer’s disease (ICD G30–G32)] or currently undergoing certain therapies (chemo/radiation therapy, dialysis, long-term ventilation) that could impede an active participation in the telemonitoring service and substantially undermine the programme’s effect. Likewise, individuals were disqualified if they were taking part in any other telemonitoring/integrated care programme [except for the COPD disease management programme (DMP)] or were not deemed suitable by the telemonitoring provider SHL Telemedizin (e.g. due to difficulties in dealing with technology or language barriers).

### Telemonitoring intervention

Patients received up to two monitoring devices [spirometer for mild to severe (FEV_1_ ≥35 %) patients and spirometer + pulse oximeter for very severe (FEV_1_ <35 %) patients] that measured vital parameters at least twice a week. Patients were free to choose the time and day of vital parameter measurement, but were called by the surveillance centre if they transferred fewer than two measurements per week. In addition, a telemonitoring console was used to answer a disease-specific [COPD assessment test (CAT)] and general well-being questionnaire (three questions) at least twice a week. Vital parameters and questionnaire data was automatically transmitted to an electronic patient record that was operated by the 24-h-available SHL surveillance centre. Moreover, users received phone calls at jointly agreed frequencies (usually every 2–3 weeks) to receive education on improved diet, exercise and lifestyle as well as support for smoking cessation. Patients were invited to contact the surveillance centre at any time should further questions occur. Based on the transmitted questionnaires and on the spirometer/pulse oximeter data, an algorithm calculated the probability of exacerbation. At enrolment, the SHL surveillance team defined measures to be taken in case of worsening health on the basis of the patient’s physician data. In the case of a high exacerbation probability, the medical staff called the patient in order to adjust emergency medication or take any other measures predefined by the physician.

### Study outcomes

The selection of the study outcomes was based on the most commonly used outcomes in the literature [8] and can be subdivided into (1) direct medical costs, (2) mortality and (3) healthcare resource utilisation. All outcomes represent the average values over the 12-month follow-up period and 24-month baseline period, respectively. COPD-related costs and healthcare utilisation were identified through the J44 diagnosis.

#### Direct medical costs

Direct medical costs for inpatient and outpatient treatment, pharmaceuticals, as well as rehabilitation, were calculated from the sickness fund’s perspective. Hospital admissions were truncated at 50,000 € per episode (first percentile) in order to limit a potential distortion by extreme outliers. From the sickness fund’s perspective, telemonitoring costs were irrelevant since programme costs were reimbursed in a profit-sharing agreement. All costs were reported in 2013 Euros.

#### Mortality

All-cause mortality was reported as the average yearly proportion of deceased individuals and hazards ratio (HR). Years of life lost (YLL) due to premature mortality were calculated by subtracting the age of death from the age- and gender-adjusted individual life expectancy [15]. We also calculated an incremental cost-effectiveness ratio (ICER) for avoiding one YLL through the use of telemonitoring. In addition, we extrapolated our mortality rates and total number of YLL to the German COPD population that would be eligible for telemonitoring (based on AOK’s eligibility criteria) in order to estimate national cost implications.

#### Healthcare resource utilisation

We compared the number of hospitalisations and outpatient physician visits (COPD-related, all-cause, ED), the (average) length of stay (COPD-related, all-cause), the proportion of hospitalised patients (all-cause, due to COPD, emergency department) and the number of pharmaceutical prescriptions between the two groups.

### Statistical analysis

In order to reduce confounding due to unbalanced baseline characteristics between the telemonitoring and control group, a two-step risk-adjustment was applied: (1) entropy balancing and (2) difference-in-difference (DiD) estimation. In a first step, we ran a reweighting algorithm (entropy balancing) in order to remove imbalances in the mean and variance of a set of pre-specified, observed covariates (e.g. age, sex, comorbidity; see “Risk adjustment” section). Entropy balancing directly recalibrates the weight of each control individual to maximise comparability to the treatment group, but at the same time it keeps the newly computed weights as close as possible to the base weights to reduce loss of information and model dependency [16]. In comparison to propensity score matching, entropy balancing achieves significantly higher covariate balance, does not discard individuals and obviates the need for manual propensity score model specification and balance checking [16]. Although balance diagnostics is not common after entropy balancing, significance tests [16] and standardised mean differences [17] were used to compare the balance of baseline characteristics before and after weighting.

In a second step, differences in outcomes between the telemonitoring and control group due to unobserved factors (e.g. undiagnosed health conditions) were minimised with the DiD estimation. The gist of DiD is to compare the difference in outcomes after (follow-up period) and before (baseline period) the intervention (telemonitoring) in the intervention group to the same difference for the control group. Outcomes in the baseline period were measured 2 years prior to the respective index date. In order to avoid biased standard errors due to serial correlation, the time series dimension of the 2-year baseline period was removed by averaging the values over 2 years and hence creating one single value per outcome measure for the baseline period [18]. The parallel trend assumption was checked by plotting relevant outcomes over time. Outcomes were calculated monthly (quarterly in the case of outpatient data, due to German reporting standards) for 2 years (baseline period) in order to verify the parallel trend over 24 data points. Finally, using the entropy weights computed in the first step, a weighted OLS regression (DiD estimator) was run with the change in costs/health outcomes as the dependent variable. In addition, the set of conditioning variables selected in the first weighting step (see “Risk adjustment” section) were used as independent variables in the weighted OLS regression in order to reduce the standard error of the treatment estimate. Because those independent variables have already been used in the entropy balancing, they have no further effect on the DiD estimator.

### Risk-adjustment

We used a set of variables that are considered to possess a high prognostic potential for the outcomes (cost, mortality and healthcare utilisation). Evidence suggests that gender, age [2, 19], comorbidities [2, 20] and pharmacy-based metrics (PBM) [21] are robust predictors of healthcare costs, mortality and resource utilisation in COPD [22]. Since comorbidities might not always be recorded through the ICD catalogue but are still treated with drugs, prescription claims data (PBM) [21] provide valuable information on the patient’s health status. Consequently, in the entropy weighting procedure, the covariates were socio-demographic variables (sex, age, and insurance status as a proxy for socio-economic status), generic comorbidity measurement instruments (29 of the total 31 Elixhauser comorbidity groups [20, 23] and 32 of the total 32 PBM groups [21]), as well as COPD-specific comorbidity measurement variables. Redundant Elixhauser and PBM groups (e.g. COPD) or those that fulfilled our exclusion criteria (e.g. metastatic cancer) were discarded. The COPD-specific group comprises indicators for COPD severity (lung function) as measured by forced expiratory volume (FEV_1_) [ICD10 GM diagnoses of J44.x0 (=FEV_1_ <35 % ≈ very severe), J44.x1 (=50 % > FEV_1_ ≥ 35 % ≈ severe), J44.x2 (=70 % > FEV_1_ ≥ 50 % ≈ moderate) or J44.x3 (=FEV_1_ ≥70 % ≈ mild)], reported tobacco addiction (ICD F17, yes/no) and membership in a COPD disease management programme (yes/no). For each patient, an ICD diagnosis was included in their risk adjustment profile if it was determined at least once in inpatient settings or at least twice within 180 consecutive days in outpatient settings. All abovementioned covariates were determined in the 2-year risk-adjustment period (variable date for telemonitoring members and 1 January 2013 for control individuals).

### Subgroup analysis

In order to detect differential treatment effects of telemonitoring for different COPD severities, we performed a separate subgroup analysis on mild to moderate COPD (FEV_1_ ≥50 %) and on severe to very severe COPD (FEV_1_ <50 %), respectively. If COPD stages of different severity existed, we chose the most severe diagnosis for the respective patient. Moreover, to analyse the effect of enrolment in a disease management programme (DMP) whilst using telemonitoring, we conducted a further subgroup analysis by DMP membership status. Because the sample composition changes in subgroup analysis, we computed new entropy weights for each subgroup.

### Sensitivity analysis

We analysed how results changed in response to (1) exclusion of deceased individuals, (2) to truncation of high-cost cases and (3) to an intention-to-treat (ITT) analysis. Owing to the fact that the last months of life often incur exceptionally high costs and healthcare utilisation, we excluded individuals who died during the intervention period and thus could have potentially distorted the effect of telemonitoring (1). In a further sensitivity analysis, we mitigated the effect of high-cost individuals by truncating the total annual costs at 50,000 € (2). Costs above this threshold are usually extreme outliers that are not representative of the entire population and might undermine true treatment effects. Finally, instead of applying an as-treated methodology, we used an intention-to-treat framework that entails the analysis of all participants regardless of their non-adherence to the assigned telemonitoring treatment protocol (3). ITT is useful in estimating the effectiveness of administering a technology in the wider community in light of inevitable treatment non-adherence [24]. Hence, we still measured outcomes at 12 months starting from telemonitoring enrolment, but we did not exclude individuals that dropped out from the telemonitoring programme during the 12-month intervention period.

## Results

**Fig. 1:**
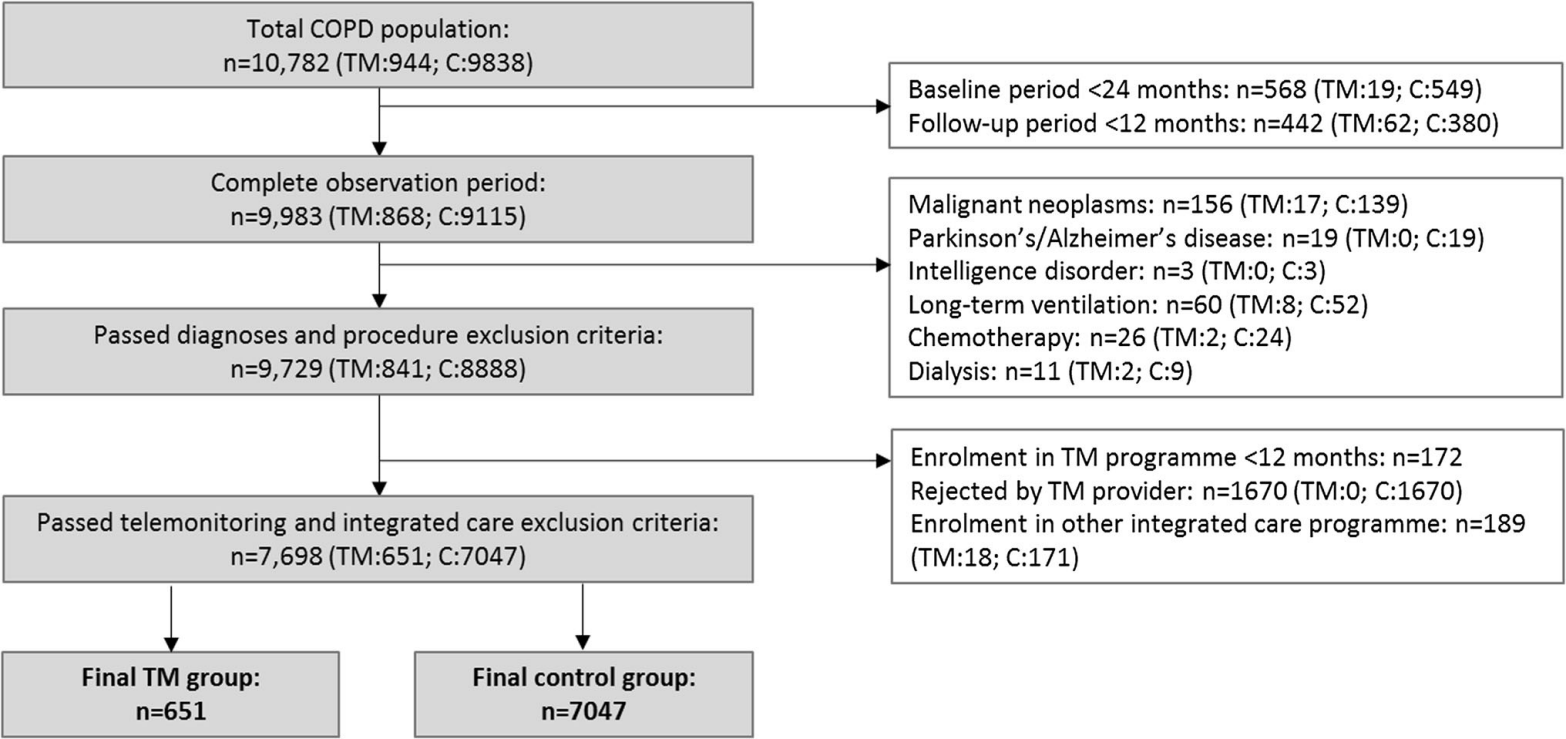
Flow-chart showing algorithm for selection of study population

Of the initial 944 telemonitoring (TM) and 9838 control individuals in the dataset, 651 and 7047 remained for the main analysis, respectively (see Fig. 1). The mean age and percentage of female participants of the telemonitoring and the control groups were 64.2 and 69.5 years and 43.9 and 49.2 %, respectively. While the proportion of patients with mild and moderate COPD was equally distributed, the intervention group had more severe (24.7 vs 17.8 %) and very severe (39.6 vs 25.2 %) cases as well as more patients with tobacco addiction (39.6 vs 23.6 %) before weighting. The average number of total Elixhauser comorbidity groups (5.2 vs 5.2) and PBM groups (6.3 vs 6.0) did not diverge importantly between the telemonitoring and control populations, respectively.

The application of entropy weighting achieved a highly balanced distribution of all observed baseline characteristics (see Table 1). While, prior to weighting, 8 out of 31 Elixhauser comorbidity groups and 10 out of 32 PBM groups differed significantly between the telemonitoring and the control groups, post weighting none of those variables showed any significant differences (see Table 5 in Appendix). Moreover, the differences in age (5.2 years, *p* < 0.001), gender (5.2 %, *p* < 0.001), tobacco addiction (16.0 %, *p* < 0.001) and COPD severity before weighting were removed to non-significant levels (all *p* = 0.999) after weighting.

**Table 1:** Baseline characteristics of the telemonitoring (TM) and control group prior to and post entropy balancing (EB)

Variables	TM	Control		D-statistic^a^		*p* value^b^	
		Before EB	After EB	Before EB	After EB	Before EB	After EB
Sample size (*N*)	651	7047		–	–	–	–
Mean age (years)	64.24	69.47	64.24	48.55	0.00	<0.05	1
Female	43.93	49.17	43.93	10.51	0.00	<0.001	1
FEV_1_ values							
FEV_1_ ≥70 %	6.91	7.25	6.91	1.32	0.00	0.81	1
70 % > FEV_1_ ≥ 50 %	17.20	17.28	17.20	0.21	0.00	1	1
50 % > FEV_1_ ≥ 35 %	24.73	17.75	24.73	17.12	0.00	<0.001	1
FEV_1_ < 35 %	39.63	25.20	39.63	31.19	0.00	<0.001	1
FEV unknown	11.52	32.51	11.52	52.35	0.00	<0.001	1
Tobacco addiction	39.63	23.64	39.63	34.90	0.00	<0.001	1
Insurance status				0.00	0.00		
Mandatory	29.03	21.77	29.03	16.74	0.00	<0.001	1
Pensionary	64.98	71.69	64.98	14.46	0.00	<0.001	1
Voluntary	5.99	6.54	5.99	2.27	0.00	0,68	1
DMP COPD enrolment	62.21	37.16	62.21	51.74	0.00	<0.001	1
Elixhauser comorbidities (see Appendix)							
Before EB	8 of 31 significantly different at *p* < 0.05						
After EB	0 of 31 significantly different at *p* < 0.05						
Pharmacy-based classes (see Appendix)							
Before EB	10 of 32 significantly different at *p* < 0.05						
After EB	0 of 32 significantly different at *p* < 0.05						

All values in % unless indicated otherwise

*EB* entropy balancing

^a^D-statistic represents the standardised mean difference

^b^ *p* value: Fisher’s exact test for dichotomous and t-test for continuous variables

### Direct medical costs

Total direct medical costs were significantly lower in the telemonitoring group (−895.11 €, *p* = 0.04). The main driver for the total cost difference was the reduction of hospitalisation costs by −1056.04 € (i = 0.01), including decreased expenses for COPD-related hospital admissions (−642.28 €, *p* < 0.001). At the same time, costs for outpatient visits slightly increased by 69.54 € (*p* = 0.05) while costs for pharmaceuticals and rehabilitation did not change significantly (Table 2).

**Table 2:** Outcomes for the telemonitoring (TM) and control group in the baseline (2 years) and follow-up period (1 year) with the respective difference-in-difference estimator and its standard error (SE)

	TM (651)		Control (7047)		DiD estimation	
	Baseline	Follow-up	Baseline	Follow-up	ATT^a^	SE
Total costs (in €)	6799	8314	6961	9371	−895*	445
Inpatient treatment	3393	4296	3768	5727	−1056**	410
Thereof due to COPD	1431	1298	1478	1987	−642***	191
Outpatient treatment	1114	1288	994	1098	70*	35
Pharmaceuticals	2120	2496	2044	2328	92	94
Rehabilitation	171	234	155	218	0	42
Indicators for healthcare utilisation						
Average length of hospital stay	6.05	4.89	5.87	6.14	−1.44***	0.34
Thereof due to COPD	4.77	2.75	4.41	4.14	−1.76***	0.29
Inpatient bed days	9.87	9.97	11.28	14.47	−3.10***	0.82
Thereof due to COPD	4.74	3.39	4.77	5.48	−2.07***	0.40
Inpatient stays	1.09	1.06	1.15	1.34	−0.21***	0.06
Thereof due to COPD	0.51	0.36	0.49	0.52	−0.18***	0.04
Thereof ED visits due to COPD	0.31	0.21	0.28	0.33	−0.14***	0.03
Proportion hospitalized (in %)	93.86	50.23	87.32	58.85	−15.16***	2.36
Thereof due to COPD	74.81	22.27	64.40	32.13	−20.27***	2.53
Thereof in ED due to COPD	49.16	14.29	40.47	22.60	−17.00***	2.47
Physician visits	15.17	16.98	13.38	13.91	1.27***	0.26
Thereof due to COPD	6.09	8.08	5.29	6.42	0.86***	0.13
Prescriptions	36.72	41.49	34.93	38.04	1.67**	0.61
Indicators for mortality						
All-cause mortality (in %)	n.a.^b^	3.23	n.a.^b^	6.22	−2.99***	n.a.

* < 0.05; ** < 0.01; *** < 0.0001

^a^Average treatment effect for the treated represents excess resource utilisation attributable to DMP

^b^Baseline values are not applicable because individuals were only eligible if alive at index date

### Mortality and ICER

During the 12-month evaluation period, a lower percentage of individuals died in the intervention group than in the control group (3.23 vs 6.22 %, *p* < 0.0001), translating into a mortality hazards ratio (HR) of 0.51 (95 % CI 0.30–0.86). Since cost savings were achieved, on average, the telemonitoring programme can be considered a dominant technology (i.e. ICER: not applicable).

Although this calculation represents a rough, probably upwards-biased approximation because the morbidity profile of those insured by AOK Bayern may not be representative for Germany, given that AOK Bayern considered 0.25 % of those it insured eligible for telemonitoring, 198.500 COPD individuals nationwide could be considered suitable for telemonitoring (0.245 % of 81.0 million). Thus, a national rollout of telemonitoring would avoid approximately 5941 deaths and 108,689 YLL per year. Given that telemonitoring reduces costs at the same time (−895.11 € per patient), cost savings of 177.7 € million could be achieved.

### Healthcare utilisation

Generally, healthcare utilisation in the telemonitoring group decreased in the inpatient sector and increased in the outpatient sector. Over the 12-month period, the proportion of patients hospitalised due to all causes (−15.16 %, *p* < 0.0001), due to COPD (−20.27 %, *p* < 0.0001) and COPD-related ED (−17.00 %, *p* < 0.0001) was consistently lower in telemonitoring patients, leading to fewer all-cause (−0.21, *p* < 0.0001), COPD-related (−0.18, *p* < 0.0001) and COPD-related ED admissions (−0.14, *p* < 0.0001). On average, people in the intervention group spent 3.1 (*p* < 0.0001) and 2.07 (*p* < 0.001) fewer days in hospital due to all causes and COPD, respectively, than the control group. The average length of stay (ALOS) declined, too. The decrease in inpatient care seems to have been compensated by more frequent outpatient visits (all-cause: 1.27, *p* < 0.0001; COPD-related: 0.86, *p* < 0.0001) and a more intense prescription of pharmaceuticals (1.67, *p* < 0.01).

### Subgroup analysis

Dividing the cohort into mild/moderate COPD (FEV_1_ ≥50 %) and into severe/very severe COPD (FEV_1_ <50 %) shows that total cost savings were larger in the less sick subgroup (mild/moderate: −1205.13 €, *p* = 0.110; severe/very severe: −518.51 €, *p* = 0.410) but differences from the control groups were not significant in both cases due to smaller sample size (see Table 3). While the biggest savings in the mild/moderate subgroup were achieved in all-cause hospitalisation costs (−1467.91 €, *p* = 0.035) through fewer all-cause hospital days (−4.3, *p* < 0.01), costs and days for COPD-related hospitalisations did not change (−23.16 €, *p* = 0.937; −0.34, *p* = 0.576). In contrast, in the severe subgroup, telemonitoring reduced COPD-related inpatient costs (−635.74, *p* = 0.018), days (−2.2, *p* < 0.0001) and ALOS (−1.81, *p* < 0.0001) but did not affect all-cause admission costs (−607.03 €, *p* = 0.290) and days (−2.0, *p* = 0.065). In both subgroups, the number of all-cause and COPD-related physician contacts significantly increased (see Table 3). Differences in mortality with a HR of 0.50 (95 % CI 0.27–0.91) were stronger in the sicker subgroup [−3.65 % (3.82 vs 7.47 %), *p* < 0.0001] than in the milder COPD group [−2.81 % (1.91 vs 4.72 %), *p* = 0.021]. The HR did not reach statistical significance in the mild/moderate population (HR 0.40, 95 % CI 0.11–1.54).

**Table 3:** Difference-in-difference estimators (ATT) and their respective standard errors (SE) for two subgroup analyses: (1) COPD severity [mild to moderate (FEV1 ≥ 50 %) and severe to very severe (FEV1 < 50 %)], and (2) DMP COPD enrolment status

	(1) Analysis by COPD severity				(2) Analysis by DMP enrolment			
	Mild to moderate (FEV_1_ ≥ 50 %) (*n*: TM = 157, C = 1729)		Severe to very severe (FEV_1_ < 50 %) (*n*: TM = 419, C = 3027)		DMP enrolment (*n*: TM = 405, C = 2619)		No DMP enrolment (*n*: TM = 246, C = 4428)	
	ATT^a^	SE	ATT^a^	SE	ATT^a^	SE	ATT^a^	SE
Total costs	−1205	748	−519	625	−886	552	−662	726
Inpatient treatment	−1468*	698	−607	573	−1051*	527	−913	643
Thereof due to COPD	−23	292	−636*	269	−649*	266	−652**	226
Outpatient treatment	160*	69	37	47	64	38	83	68
Pharmaceuticals	24	78	84	161	141	112	98	131
Rehabilitation	79	99	−32	50	−40	48	69	79
Indicators for healthcare utilisation								
Average length of hospital stay	−1.59*	0.68	−1.23**	0.46	−1.79***	0.45	−1.09*	0.55
Thereof due to COPD	−0.71	0.56	−1.81***	0.36	−2.03***	0.38	−1.44**	0.45
Inpatient bed days	−4.30**	1.44	−2.03	1.10	−3.00**	1.09	−2.90*	1.28
Thereof due to COPD	−0.34	0.60	−2.22***	0.55	−2.40***	0.53	−1.65**	0.58
Inpatient stays	−0.30**	0.11	−0.11	0.09	−0.19*	0.09	−0.26**	0.10
Thereof due to COPD	−0.06	0.06	−0.17**	0.05	−0.20***	0.05	−0.17**	0.05
Thereof ED visits due to COPD	−0.09*	0.04	−0.13**	0.04	−0.14***	0.04	−0.17***	0.04
Proportion hospitalised (in %)	−9.58*	4.83	−12.84***	3.00	−16.65***	3.13	−14.99***	3.62
Thereof due to COPD	−11.81*	5.29	−19.80***	3.25	−21.51***	3.38	−20.18***	3.86
Thereof in ED due to COPD	−10.71*	4.87	−17.40***	3.22	−17.57***	3.35	−18.08***	3.62
Physician visits	1.55**	0.55	1.10***	0.32	1.21***	0.34	1.35***	0.41
Thereof due to COPD	0.82***	0.25	0.89***	0.17	0.78***	0.18	0.85***	0.19
Prescriptions	2.67*	1.14	1.31	0.79	1.03	0.78	2.75**	0.97
Indicators for mortality								
All-cause mortality (in %)	−2.81*	n.a.	−3.65***	n.a.	−3.26***	n.a.	−2.31	n.a.

The second subgroup analysis revealed that DMP membership did not prominently affect the magnitude or direction of the effect of telemonitoring on costs and other outcomes. Cost-savings for all-cause (DMP: −1051 €; non-DMP: −913 €) and COPD-related hospital admissions (DMP: −649 €; non-DMP: −652 €) was similar in both groups, although statistical significance for all-cause admissions was only reached in the DMP group. No clinically important differences were observed for indicators of healthcare utilisation between the DMP and non-DMP populations. Mortality HRs were still in favour of the telemonitoring interventions in both DMP groups (DMP: HR 0.40, 95 % CI 0.18–0.86; non-DMP: HR 0.67, 95 % CI 0.32–1.40) but was not significant in the non-DMP arm.

### Sensitivity analysis

In all three sensitivity analysis scenarios [(1) excluding dead individuals, (2) truncation, (3) ITT], telemonitoring was 13.28–38.15 % less effective in reducing total costs than in the baseline scenario (see Table 4) and the differences lost statistical significance [(1) −776.26 €, *p* = 0.074; (2) −553.62 €, *p* = 0.132; (3) −706.30 €, *p* = 0.089]. However, the reductions in all-cause [(1: excluding dead): −936.43 €, *p* = 0.019; (2: truncation): −826.14 €, *p* = 0.020; (3: ITT): −919.54 €, *p* = 0.014] and COPD-related inpatient costs [(1: excluding dead): −624.71 €, *p* = 0.001; (2: truncation): −597.94 €, *p* = 0.001; (3: ITT): −554.96 €, *p* = 0.003] remained significant and stable in all scenarios. Relative changes to baseline in all-cause and COPD-related costs ranged from 11.33 to 21.77 % and from 2.74 to 13.60 %, respectively. For scenarios (1: excluding dead) and (3: ITT), direction, magnitude and significance of differences in healthcare utilisation continued to be very similar to the baseline scenario. The mortality hazards ratio further declined in favour of telemonitoring in the (3) ITT analysis (HR 0.40, 95 % CI 0.24–0.67).

**Table 4:** Sensitivity analysis: three scenarios (1: excluding dead, 2: cost truncation, 3: intention-to-treat) with difference-in-difference estimators (ATT) and their relative change compared to the baseline scenario

	Excluding dead (1) (*n*: TM = 630, C = 6607)		Cost truncation (2) (*n*: TM = 651, C = 7047)		ITT (3) (*n*: TM = 815, C = 7047)		Baseline
	ATT^a^	Δ %	ATT^a^	Δ %	ATT^a^	Δ %	ATT^a^
Total costs	−776.26	13.28	−553.62	38.15	−706.30	21.09	−895.11*
Inpatient treatment	−936.43*	11.33	−826.14*	21.77	−919.54*	12.93	−1056.04**
Thereof due to COPD	−624.71***	2.74	−597.94**	6.90	−554.96**	13.60	−642.28***
Outpatient treatment	63.98	7.99	68.82*	1.03	65.11*	6.36	69.54*
Pharmaceuticals	101.71	−10.79	130.19	−41.82	145.13	−58.09	91.80
Medical appliances/rehabilitation	−5.51	−1266	−0.40	0.00	2.99	−642	−0.40
Indicators for healthcare utilisation							
Average length of hospital stay	−1.48***	−2.91	n.a.	n.a.	−1.24***	13.64	−1.44***
Thereof due to COPD	−1.72***	2.18	n.a.	n.a.	−1.68***	4.673	−1.76***
Inpatient bed days	−3.01***	2.72	n.a.	n.a.	−2.82***	9.05	−3.10***
Thereof due to COPD	−2.07***	−0.21	n.a.	n.a.	−1.86***	10.03	−2.07***
Inpatient stays	−0.21**	4.12	n.a.	n.a.	−0.16**	26.04	−0.21***
Thereof due to COPD	−0.19***	−4.95	n.a.	n.a.	−0.15***	14.03	−0.18***
Thereof ED visits due to COPD	−0.15***	−3.75	n.a.	n.a.	−0.13***	10.50	−0.14***
Proportion hospitalised (in %)	−14.95***	1.38	n.a.	n.a.	−11.77***	22.36	−15.16***
Thereof due to COPD	−19.98***	1.42	n.a.	n.a.	−18.86***	6.95	−20.27***
Thereof in ED due to COPD	−16.72***	1.64	n.a.	n.a.	−15.12***	11.06	−17.00***
Physician visits	1.09***	14.82	n.a.	n.a.	1.21***	5.10	1.27***
Thereof due to COPD	0.77***	10.03	n.a.	n.a.	0.76***	11.85	0.86***
Prescriptions	0.91	45.35	n.a.	n.a.	1.48**	11.31	1.67**
Indicators for mortality							
All-cause mortality (in %)	n.a.	n.a.	n.a.	n.a.	−3.73***	−24.58	−2.99***

Δ %: deviation (in %) of respective sensitivity analysis value from baseline scenario value

* < 0.05; ** < 0.01; *** < 0.0001

^a^Average treatment effect for the treated represents excess resource utilisation attributable to DMP

## Discussion

We demonstrated in this observational, population-based cohort study that our 12-month telemonitoring intervention for COPD entails a strong reduction in mortality (HR 0.51, 95 % CI 0.30–0.86), in total yearly costs by −895.11 €, driven by substantial savings in hospitalisation costs (−1056.04 €), and in inpatient healthcare utilisation. Costs (69.54 €) and number of outpatient visits (1.27) slightly increased, though. In terms of ICER, telemonitoring is a dominant technology compared to standard care.

The most striking finding in this study is the marked positive impact telemonitoring had on mortality at 12 months (3.23 vs 6.22 %, *p* < 0.0001; HR 0.51, 95 % CI 0.30–0.86). The largest RCT in telemonitoring, the Whole System Demonstrator (WSD) project, found a very similar mortality HR of 0.59 (95 % CI 0.43–0.80) with somewhat higher mortality figures (4.6 vs 8.3 %) [25]. Direct comparisons must be treated with caution, though, because the WSD recruited diabetes and heart failure patients in addition to COPD patients. None of the meta-analyses [8–10] and systematic reviews [11] found any overall statistically significant effect on mortality, potentially because most of the included studies were underpowered (total median sample size: 70) to specifically detect a mortality difference. Meta-analytic evidence from better studied diseases, in particular CHF, indicates similar reductions in mortality risk, ranging between 34 and 20 % [6].

Moreover, the clear decline in hospitalisations found in our study is corroborated in the literature. Two meta-analyses [8, 9] and two systematic reviews [10, 11] concluded that telemonitoring reduced the risk of hospital admission, with pooled odds ratios (OR) and risk ratios (RR) ranging between OR 0.46 [8] and RR 0.72 [9]. The reduction in the proportion of people hospitalised due to COPD (−20.27 %) and admitted to ED due to COPD (−17.00 %) indicates that the telemonitoring intervention might reduce the number of severe exacerbations and, hence, the need for emergency hospital care. Other studies reported similar, absolute reductions in proportions of individuals with ED visits of 19 % [26] and 23 % [27]. Indeed, the literature suggests that telemonitoring can decrease the number of exacerbations [28], which are most commonly associated with a worsening of peripheral oxygen saturation [9].

Although the exact mechanisms of reducing hospitalisations is not completely clear in our study, we suspect two possible pathways: first, it is possible that the monitoring of patients’ oxygen saturation and weight can predict a worsening of the health state to some extent. However, the correlation between daily variation in spirometry and other physiological measures and exacerbations is still poorly understood, leading to a high rate of false-positive warnings [29]. Machine-learning algorithms—taking into account a wider array of variables, such as physiological signs, symptoms, disease severity, prior hospitalisations, medication intake, demographic characteristics as well as indicators for depression, anxiety or social isolation—could boost telemonitoring’s predictive power in detecting exacerbations [29]. Second, patients in our programme received support and education on correct disease management, potentially allowing them to spot a COPD-related worsening of their health in a more timely manner. It is possible that patients learned to better adhere to their medication regimen and, if they perceived the need, to initiate pharmacological therapy with β_2_-adrenergic agonists or corticosteroids. A tendency for increased spending on medication (+92 €) as well as evidence on the positive effect of self-management on medication intake in COPD [30] support our hypothesis. Early patient recognition of exacerbations and prompt treatment initiation are associated with reduced risk of hospitalisation and faster exacerbation recovery [31]. Both reduced risk of hospitalisation and faster exacerbation recovery were also found in our study, manifesting themselves in a reduced proportion of patients with hospitalisations (−15.16 %) and a shorter length of hospital stay (−1.44 days) in the intervention group. This finding might suggest that individuals using telemonitoring are hospitalised with less severe exacerbations, potentially because they were recognised and treated earlier.

Given the reductions in frequency and duration of hospitalisations, which constituted 51 and 61 % of the total costs in the follow-up period of intervention and control group, respectively, overall costs were considerably lower in the telemonitoring arm (−895.11 €). Savings in all-cause and COPD-related hospital costs were insensitive to model specifications and analysis methodologies. The decrement in inpatient care seems to have been compensated by higher use of outpatient services (69.54 €). Direct comparisons with other cost studies can hardly be drawn as the telemonitoring technology itself as well as health system-specific reimbursement may largely vary. Still, most studies with a cost-assessment reported savings between 12 and 17 % in the telemonitoring group [32], which is similar to the reduction of 11 % in the follow-up period of our cohort.

Although irrelevant in this specific profit-sharing agreement between the sickness fund and telemonitoring provider, we underestimated the true costs of telemonitoring because we did not possess any information on the costs of the programme (including investments and operating costs for software, hardware, personnel, administration). Consequently, it might take a few years until cost-savings from less intense healthcare use compensate for the technology investment. Given yearly telemonitoring fees of 677 € found in a Danish study [33], the sickness fund would still save 218 € (=895–677) while still reducing mortality. Even at a yearly telemonitoring service cost of 1000 € and a resulting increase in expenditure of 105 € (=895–1000), the ICER would be highly cost-effective with 191 € per life-year gained.

The subgroup analysis revealed that patients with (very) severe COPD experienced greater reductions in mortality as well as in cost, number and duration of COPD-related hospitalisations than individuals with mild/moderate COPD. This indicates, again, that telemonitoring may effectively decrease the number of exacerbations that require inpatient treatment. Because high-risk patients are usually hospitalised more frequently, they have a greater baseline potential for cutting hospitalisations and costs. A high-quality RCT corroborated our findings, showing that telemonitoring was less effective in curbing hospitalisation rates for mild cases than for severe ones [34]. Similarly, a study on telemonitoring in asthma found no improvements in health outcomes in individuals with mild disease, but showed a reduced risk of admission to hospital for high-risk patients [35]. While savings in all-cause hospitalisations were considerable in the mild/moderate group (−1468 €, *p* < 0.05), the cost reduction in COPD-related cost was not significant. Potential reasons for a lack of statistically and clinically significant changes could be the small sample size (TM; *n* = 157) as well as the fact that COPD-related hospital costs constitute only roughly 28 % of total inpatient costs in our mild/moderate sample. In line with our data, the literature indicates that comorbidities, such as ischemic heart failure or diabetes, are more important drivers of hospitalisation costs in these patients [36]. A positive spill-over effect of TM on the management of concurrent diseases might be possible.

Another important finding of the subgroup analysis is that telemonitoring continues to be cost-saving for COPD-related hospitalisations, reduces healthcare utilisation and still displays a trend for reduced mortality, even when isolating its effect from additional interventions in usual care, such as disease management programmes (DMPs). The lack of statistical significance in some outcomes is most likely due to decreased sample size, as controlling for DMP participation in the baseline scenario still delivered significant results. In most published studies, it was impossible to disentangle the effect of telemonitoring from usual care because the intervention group received enhanced clinical care that could affect outcomes on its own. For instance, care enhanced through the German DMPs for COPD have been found to improve clinical outcomes [37]. A recent randomised controlled trial (RCT) in the UK, however, disentangled the effects of telemonitoring from the effect of the remaining elements of healthcare service and concluded that telemonitoring was not effective in reducing rates of/time to admission, neither QoL [34]. The reasons for these diverging findings could be rooted in differences in telemonitoring interventions employed, as well as in the provision of standard care.

### Limitations

Our results should be interpreted in light of certain data-related and methodological limitations. First, our administrative data provide only limited information on the clinical progression of disease and on smoking status, which are both predictors of health and cost outcomes [1, 2]. Although COPD severity can be approximated in our data by the fourth and fifth digits of the ICD code (J44.XX), clinicians often do not precisely specify these digits in everyday practice. Nor does our data indicate whether telemonitoring simply shifts the burden and costs of care away from the inpatient sector towards the patients themselves or towards their family members and caregivers. Moreover, we had no information on causes of death which would have allowed disentangling the effect of DMP on all-cause and COPD-specific mortality. Similarly, we did not possess life-tables for COPD populations to calculate the number of life-years gained. By using life-tables from the general population, we might overestimate the number of life-years gained for the telemonitoring group. Overestimation is also a potential issue in the budget impact analysis, because the AOK Bayern insured population might be sicker than the average German population, hence inflating the percentage of patients eligible for the TM. In addition, we might have underestimated the number of outpatient physician visits in both groups due to German medical coding modalities and reporting standards. The last data-related limitation is the fact that we had no data to adjust for potentially diverging treatment intensity in the TM group. Despite regularly scheduled remote health examinations, some patients might have participated with a higher adherence to the programme than others. Second, the inferences from the entropy balancing in this non-randomised study rely on the assumption that all relevant patient-related covariates have been included and that no unobserved confounders exist (‘unconfoundedness assumption’) [38]. This assumption is not empirically testable because it is impossible to measure hidden confounders. For instance, COPD patients participating in the telemonitoring programme might be more motivated to address their disease, have a healthier lifestyle or more social support than those who did not enrol. In particular, the final inclusion of the patients into the TM programme was within the discretion of the TM provider, introducing a potential source of selection bias. However, we minimised the impact of potential hidden confounders by constructing a DiD estimation framework, which even accounts for unobserved differences. Moreover, we conducted an extensive sensitivity analysis to verify the robustness of our results.

## Conclusion

This is the first German study to demonstrate that telemonitoring for COPD is a viable technology that reduces mortality, healthcare costs and utilisation at 12 months. Contrary to widespread fear, lowering the intensity of care does not seem to impact unfavourably on health outcomes. Subgroups with severe COPD benefit more from the technology than patients with lighter forms of the disease. It remains to be seen, however, whether these positive results are constant over a longer observation period. Future improvements in predicting exacerbations through more powerful algorithms and the use of wearable and mobile devices will underpin the case for a system-wide implementation of telemonitoring for COPD. It should be stressed, however, that telemonitoring alone will not suffice in providing high-quality treatment for COPD patients. Instead, telemonitoring should be introduced as a supporting component of integrated case management, which approaches COPD and its comorbidities holistically.

## Appendix

See Table 5.

**Table 5:** Elixhauser comorbidity groups, pharmacy-based metrics and other (disease-specific) variables before and after entropy balancing with balance statistics

	TM (in %)	Control (in %)		*p* value^a^		D-statistic^b^	
		Pre	Post	Pre	Post	Pre	Post
Elixhauser comorbidity groups							
(1) Congestive heart failure	31.49	36.17	31.49	<0.05	1	0.10	0.00
(2) Cardiac arrhythmias	19.97	25.43	19.97	<0.01	1	0.13	0.00
(3) Valvular disease	9.83	11.18	9.83	0.3272	1	0.04	0.00
(4) Pulmonary circulation disorders	9.22	10.00	9.22	0.5837	1	0.03	0.00
(5) Peripheral vascular disorders	22.43	21.58	22.43	0.6191	1	0.02	0.00
(6) Hypertension uncomplicated	72.20	74.37	72.20	0.2239	1	0.05	0.00
(7) Hypertension complicated	17.97	22.08	17.97	<0.05	1	0.10	0.00
(8) Paralysis	2.76	2.95	2.76	0.9034	1	0.01	0.00
(9) Other neurological disorders	3.99	3.89	3.99	0.833	1	0.01	0.00
(10) Chronic pulmonary disease	99.69	92.17	99.69	<0.001	1	0.39	0.00
(11) Diabetes uncomplicated	31.18	33.09	31.18	0.3377	1	0.04	0.00
(12) Diabetes complicated	13.21	14.52	13.21	0.382	1	0.04	0.00
(13) Hypothyroidism	18.43	17.17	18.43	0.4165	1	0.03	0.00
(14) Renal failure	13.06	19.74	13.06	<0.001	1	0.18	0.00
(15) Liver disease	21.51	18.93	21.51	0.1182	1	0.06	0.00
(16) Peptic ulcer disease excluding bleeding	2.46	2.26	2.46	0.6811	1	0.01	0.00
(17) AIDS/HIV	0.15	0.06	0.15	0.3572	1	0.03	0.00
(18) Lymphoma	0.00	0.00	0.00	–	1	–	–
(19) Metastatic cancer	0.00	0.00	0.00	–	–	–	–
(20) Solid tumor without metastasis	0.15	0.06	0.15	0.3572	1	0.03	0.00
(21) Rheumatoid arthritis	7.83	6.87	7.83	0.3347	1	0.04	0.00
(22) Coagulopathy	1.69	1.43	1.69	0.6059	1	0.02	0.00
(23) Obesity	31.80	24.98	31.80	<0.001	1	0.15	0.00
(24) Weight loss	4.76	4.19	4.76	0.4762	1	0.03	0.00
(25) Fluid and electrolyte disorders	27.80	30.41	27.80	0.1807	1	0.06	0.00
(26) Blood loss anemia	0.77	0.68	0.77	0.8021	1	0.01	0.00
(27) Deficiency anemias	4.45	5.12	4.45	0.5135	1	0.03	0.00
(28) Alcohol abuse	11.67	7.92	11.67	<0.01	1	0.13	0.00
(29) Drug abuse	3.38	2.27	3.38	0.0794	1	0.07	0.00
(30) Psychoses	2.30	2.23	2.30	0.8895	1	0.01	0.00
(31) Depression	34.41	28.93	34.41	<0.01	1	0.12	0.00
Pharmacy-based groups							
(1) Antiplatelet	7.83	8.36	7.83	0.7106	1	0.02	0.00
(2) Anticoagulant	10.91	14.69	10.91	<0.01	1	0.11	0.00
(3) Epilepsy	10.29	9.21	10.29	0.3593	1	0.04	0.00
(4) Hypertension	18.59	19.74	18.59	0.5029	1	0.03	0.00
(5) HIV	0.15	0.10	0.15	0.507	1	0.02	0.00
(6) Tuberculosis	0.31	0.30	0.31	1	1	0.00	0.00
(7) Rheumatic conditions	64.06	46.20	64.06	<0.001	1	0.36	0.00
(8) Hyperlipidemia	34.56	32.60	34.56	0.3156	1	0.04	0.00
(9) Malignancies	0.31	0.27	0.31	0.6965	1	0.01	0.00
(10) Parkinson’s disease	2.00	2.51	2.00	0.5088	1	0.03	0.00
(11) Renal disease	0.61	0.92	0.61	0.6609	1	0.04	0.00
(12) End stage renal disease (ESRD)	0.31	0.44	0.31	1	1	0.02	0.00
(13) Anti-arrhythmic	2.00	6.29	2.00	<0.001	1	0.22	0.00
(14) Ischemic heart disease/angina	7.07	9.47	7.07	<0.05	1	0.09	0.00
(15) Congestive heart failure	66.67	70.71	66.67	<0.05	1	0.09	0.00
(16) Diabetes	20.74	21.14	20.74	0.841	1	0.01	0.00
(17) Glaucoma	3.53	4.56	3.53	0.2746	1	0.05	0.00
(18) Liver failure	0.31	0.71	0.31	0.3184	1	0.06	0.00
(19) Acid peptic disease	62.67	56.62	62.67	<0.01	1	0.12	0.00
(20) Transplantation	0.92	0.75	0.92	0.635	1	0.02	0.00
(21) Respiratory illness, asthma	98.62	83.77	98.62	<0.001	1	0.54	0.00
(22) Thyroid disorders	22.12	19.98	22.12	0.2017	1	0.05	0.00
(23) Gout	15.51	16.62	15.51	0.5081	1	0.03	0.00
(24) Inflammatory bowel disease, chronic	1.23	0.70	1.23	0.1453	1	0.05	0.00
(25) Pain and inflammation	61.14	55.54	61.14	<0.01	1	0.11	0.00
(26) Pain	19.66	17.95	19.66	0.2872	1	0.04	0.00
(27) Depression	30.72	24.66	30.72	<0.001	1	0.14	0.00
(28) Psychotic illness	6.61	5.35	6.61	0.1762	1	0.05	0.00
(29) Bipolar disorders	0.31	0.21	0.31	0.6501	1	0.02	0.00
(30) Anxiety and tension	11.67	11.31	11.67	0.796	1	0.01	0.00
(31) Hepatitis	0.00	0.09	0.00	1	1	0.04	0.00
(32) Ischemic heart disease	51.15	54.99	51.15	0.0641	1	0.08	0.00
Other matching variables							
Female (in %)	43.93	49.17	43.93	<0.05	1	0.11	0.00
Age	64.24	69.47	64.24	<0.001	1	0.49	0.00
Tobacco addiction	39.63	23.64	39.63	<0.001	1	0.35	0.00
FEV_1_ values							
FEV_1_ ≥ 70 %	6.91	7.25	6.91	0.8125	1	0.01	0.00
70 % > FEV_1_ ≥ 50 %	17.20	17.28	17.20	1	1	0.00	0.00
50 % > FEV_1_ ≥ 35 %	24.73	17.75	24.73	<0.001	1	0.17	0.00
FEV_1_ < 35 %	39.63	25.20	39.63	<0.001	1	0.31	0.00
FEV unknown	11.52	32.51	11.52	<0.001	1	0.52	0.00
DMP enrolment	62.21	37.16	62.21	<0.001	1	0.52	0.00
Insurance status							
Mandatory	29.03	21.77	29.03	<0.001	1	0.17	0.00
Pensionary	64.98	71.69	64.98	<0.001	1	0.14	0.00
Voluntary	5.99	6.54	5.99	0.6774	1	0.02	0.00
